# A novel rat model of ovarian cancer and cisplatin chemotherapy-related cognitive impairment

**DOI:** 10.1016/j.bbih.2026.101373

**Published:** 2026-09-22

**Authors:** Naomi Lomeli, Diana C. Pearre, Javier J. Lepe, Donovan A. Argueta, Mya A. Arellano, Kalpna Gupta, Thomas H. Taylor, Daniela A. Bota

**Affiliations:** a Department of Neurology, University of California Irvine, Irvine, CA, USA; b Gynecologic Oncology, Providence Cancer Institute, Burbank, CA, USA; c Department of Medicine, Division of Hematology/Oncology, University of California, Irvine, Irvine, CA, USA; d Division of Hematology, Oncology and Transplantation, School of Medicine, University of Minnesota, Minneapolis, MN, USA; e Department of Pharmaceutical Science, University of California, Irvine, CA, USA; f Chao Family Comprehensive Cancer Center, University of California Irvine, Irvine, CA, USA

**Keywords:** Cisplatin (CDDP), Chemotherapy, Hippocampus, Ovarian cancer, Cytokines, Inflammation, Cognitive function, RNU rats

## Abstract

Cancer-related cognitive impairment (CRCI) is a common, persistent complication of cancer and its treatment, yet its etiology in ovarian cancer remains poorly understood. Preclinical rat models integrating tumor burden and cognitive outcomes in ovarian cancer remain limited, and the contribution of tumor-associated processes has been insufficiently characterized. We developed an SKOV3.ip1 human ovarian cancer xenograft model of CRCI in female RNU rats, combined with a clinically relevant cisplatin regimen (4 cycles, 5 mg/kg, i.p.). Animals were randomly assigned to non-tumor (NT) or ovarian tumor-bearing (OvT) groups and to receive cisplatin (CDDP) or vehicle (VEH), resulting in four groups: NT + VEH, NT + CDDP, OvT + VEH, and OvT + CDDP. Anxiety-like behavior was assessed using the open field test, and cognitive function was evaluated using context object discrimination and novel object recognition tasks. Serum cytokine levels were measured in relation to behavioral outcomes. CDDP significantly impaired novel object recognition in non-tumor-bearing animals, but not in tumor-bearing animals which were impaired in object recognition regardless of treatment. NT + CDDP, OvT + VEH, and OvT + CDDP animals were significantly impaired relative to NT + VEH. Tumor implantation and cisplatin exposure were associated with alterations in systemic cytokine levels. Exploratory analyses identified IL-10 and C-reactive protein as candidate markers of reduced cognitive performance, although overall discriminative performance was modest. These findings demonstrate that the cognitive effects of chemotherapy depend on tumor status and underscore the importance of incorporating tumor-bearing conditions into preclinical CRCI models. Our model provides a translational platform to investigate tumor-treatment interactions and explore candidate inflammatory markers associated with cognitive outcomes in ovarian cancer.

## Highlights

• SKOV3.ip1 xenograft in RNU rats is the first ovarian cancer model of CRCI.
• Cisplatin-induced cognitive impairment depends on tumor status.
• Systemic levels of IL-10 and CRP are prognostic for reduced cognitive functioning.

## 1 Introduction

Ovarian cancer is the eighth most common cancer in women, with more than 21,000 individuals diagnosed annually in the United States (American Cancer Society, 2026). Cognitive and mood changes associated with cancer and its treatment are common and can persist for years after treatment completion, significantly impacting quality of life (QoL) (Janelsins et al., 2014; Zhang et al., 2025). Over 70% of patients with gynecological cancer experience cancer-related cognitive impairments (CRCI, also referred to as “chemobrain”), with more than 35% of survivors experiencing persistent cognitive deficits in executive function, processing speed, attention, concentration, and verbal memory, as well as depression and anxiety (De Rosa et al., 2021; Correa et al., 2017; Mayo et al., 2021). Despite this high prevalence, CRCI in patients with ovarian cancer remains understudied, and both pre-clinical and clinical investigations examining the underlying mechanisms are limited (Pearre and Bota, 2018; Correa and Hess, 2012). Platinum-based chemotherapy (cisplatin [CDDP] or carboplatin) remains the first-line treatment for many gynecologic cancers. While platinum-based chemotherapy is recognized as a contributor to CRCI, accumulating evidence indicates that tumor-related biological processes may independently influence cognition and affective functioning, even prior to treatment initiation (Mampay et al., 2021).

Evidence of cognitive impairment prior to treatment initiation has been reported across multiple non-central nervous system (CNS) cancers, supporting a role for tumor-associated processes in CRCI (Schrepf et al., 2015). In ovarian cancer, emerging data suggest baseline deficits in delayed recall and visuospatial and executive function, although studies are limited by small sample sizes and a lack of standardized neuropsychological paradigms across studies (Wang et al., 2024). Beyond pre-treatment deficits, longitudinal studies have demonstrated substantial cognitive decline during and after cancer treatment, with persistent impairments across multiple cognitive domains, including decreased attention, processing speed, and reaction time (Hess et al., 2010, 2015). Neuroimaging studies have also identified reduced gray matter volume and altered prefrontal cortex activity following treatment (Correa et al., 2017; McDonald and Saykin, 2013). In addition to cognitive deficits, patients with ovarian cancer experience anxiety and depression, which have been associated with elevated circulating inflammatory cytokines, signifying biological links between systemic tumor burden and mood-related outcomes (Lutgendorf et al., 2008a, 2013).

Chronic systemic inflammation has emerged as a key biological mechanism linking peripheral tumor burden to alterations in brain structure, cognition, and mood. Elevated circulating cytokines may drive neuroinflammation by signaling across the blood-brain barrier and have been associated with impaired cognitive performance across multiple cancer populations (Lutgendorf et al., 2008a, 2008b; Lyon et al., 2016). In preclinical models of CRCI, neuroinflammatory processes are accompanied by microglial activation, reduced neurogenesis, and synaptic alterations that parallel cognitive and mood-related deficits (Gibson et al., 2019; Andres et al., 2014). Given the emerging evidence of tumor-associated neuroinflammation, there is a critical need for preclinical tumor-bearing animal models to investigate these mechanisms, as most CRCI models have focused primarily on chemotherapy-induced neurotoxicity in the absence of tumor burden (Schrepf et al., 2015).

Our prior work in Sprague Dawley rats characterized cisplatin-induced cognitive dysfunction in the absence of tumor burden, identifying mitochondrial dysfunction as a key contributor, an effect mitigated by the antioxidant N-acetylcysteine (NAC) (Lomeli et al., 2017), and reductions in brain-derived neurotrophic factor (BDNF) as an additional mechanism (Lomeli et al., 2024). However, preclinical ovarian cancer models incorporating both tumor burden and cognitive outcomes remain limited. To address this gap, we established the SKOV3.ip1 human ovarian tumor model in female athymic nude rats (Cr:NIH-RNU), incorporating a clinically relevant cisplatin regimen and assessing cognitive function, systemic serum cytokine levels, and survival. This model was recently applied to evaluate the neuroprotective efficacy and oncologic safety of NAC (Lomeli et al., 2025); however, its methodological framework has not been comprehensively described. Here, we present a detailed methodological characterization of our model, including tumor engraftment characteristics, cohort attrition, survival outcomes, systemic cytokine profiling, and correlations with behavioral outcomes, to facilitate its use in mechanistic and therapeutic studies of CRCI.

## 2 Methods

### 2.1 Animals

All animal studies were approved by the Institutional Animal Care and Use Committee (IACUC; AUP-22-097) of the University of California, Irvine, and were conducted in accordance with the National Institutes of Health (NIH) guidelines for the care and use of laboratory animals. A total of 88 female athymic nude rats (Cr:NIH-RNU; Charles River), aged three to four months, were used in this study. Animals were housed in groups of two to four rats per cage under a standard 12 h light/dark cycle (lights off at 18:30), with controlled temperature (23°C ± 1°C) and humidity (45 ± 10%), and provided *ad libitum* access to standard rodent chow diet (Envigo Teklad 2920X) and water. Rats were acclimated to the vivarium for one week and habituated to handling three times per week prior to tumor or vehicle induction.

Animals were randomly assigned into four experimental groups: non-tumor-bearing rats receiving vehicle (NT + VEH) or cisplatin (NT + CDDP), and ovarian tumor-bearing rats receiving vehicle (OvT + VEH) or cisplatin (OvT + CDDP). All efforts were made to minimize animal suffering and to minimize the total number of rats used. This study was conducted across three separate experimental cohorts, and data from all cohorts were pooled for analysis (Fig. 1).

**Fig. 1:**
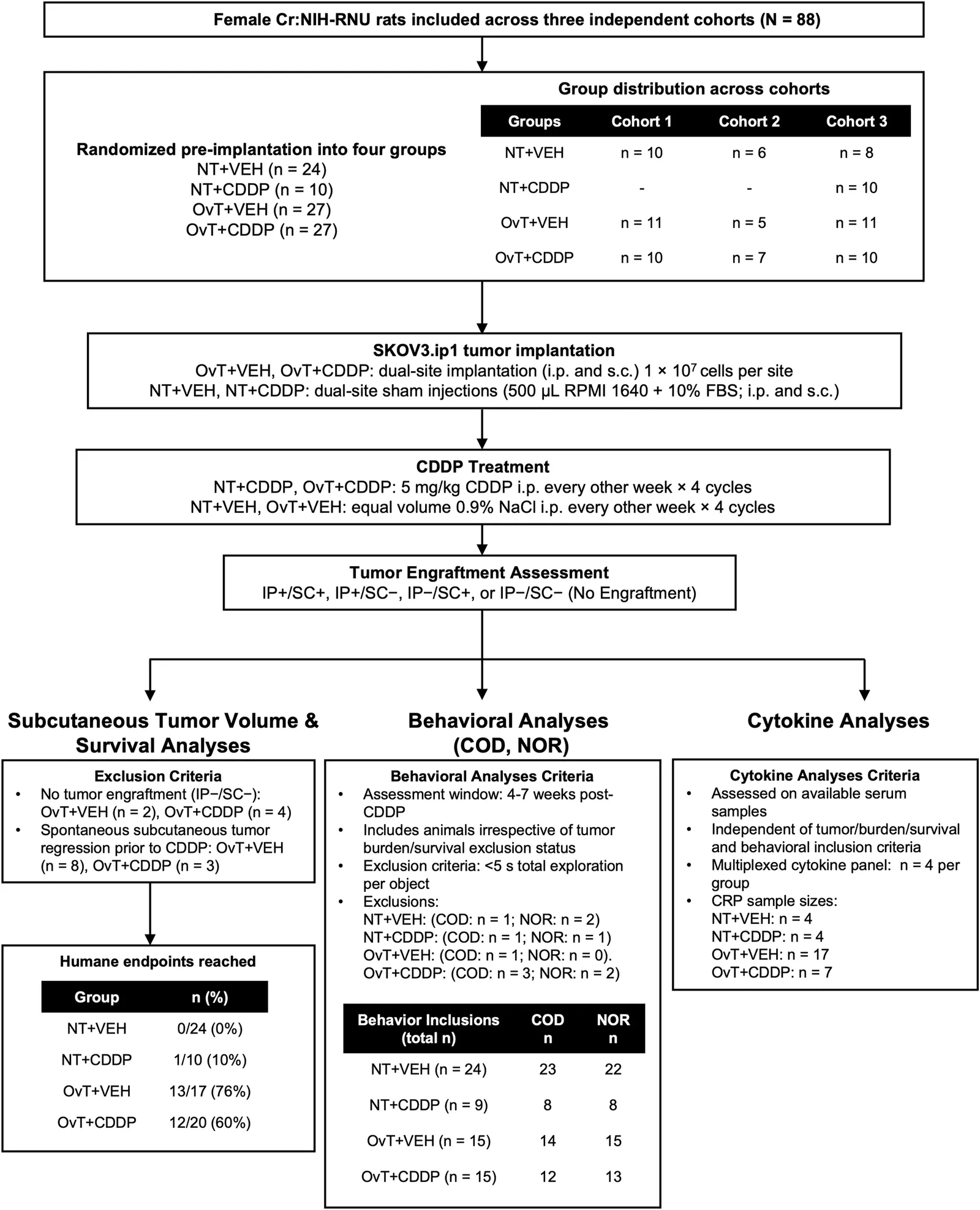
**Study design, engraftment outcomes, and analytic inclusion criteria flowchart.** A total of N = 88 female Cr:NIH-RNU rats were included across three independent experimental cohorts (Cohorts 1-3) and randomized pre-implantation into four groups: NT + VEH (n = 24), NT + CDDP (n = 10), OvT + VEH (n = 27), and OvT + CDDP (n = 27). Ovarian tumor-bearing groups (OvT + VEH and OvT + CDDP) received dual-site SKOV3.ip1 implantation (i.p. and s.c.; 1 × 10^7^ cells per site), whereas NT groups received dual-site sham injections (500 μL RPMI 1640 + 10% FBS). Cisplatin (5 mg/kg, i.p.) or an equal volume of 0.9% NaCl was administered every other week for four cycles. Tumor engraftment (IP+/SC+, IP+/SC−, IP−/SC+, IP−/SC−) was assessed prior to analysis. Animals failing engraftment or exhibiting spontaneous subcutaneous tumor regression were excluded from subcutaneous tumor volume and survival analyses. Humane endpoints were defined as ≥ 20% body weight loss or tumor size limits (tumor diameter ≥ 3.5 cm). Behavioral analyses were performed on animals that completed treatment and survived to at least four weeks post-treatment completion, regardless of tumor engraftment status or survival analysis exclusions, with task-specific exclusions applied. Serum cytokine profiling was performed on available samples, independent of tumor engraftment status, survival, and behavioral inclusion criteria. Abbreviations: CDDP, cisplatin; COD, context object discrimination; IP, intraperitoneal; NOR, novel object recognition; NT, non-tumor-bearing; OvT, ovarian tumor-bearing; SC, subcutaneous; VEH, vehicle.

### 2.2 SKOV3.ip1 ovarian tumor induction

The human SKOV3.ip1 line, derived from the parental SKOV3 human epithelial ovarian adenocarcinoma cell line, was maintained in RPMI 1640 supplemented with 10% fetal bovine serum (FBS) in a humidified incubator at 37°C with 5% CO_2_. SKOV3.ip1 was selected based on a pilot engraftment study in which four human ovarian cancer cell lines (TOV21G, OVCAR3, OVCAR8, and SKOV3.ip1) were implanted at dual sites (intraperitoneal and subcutaneous in RNU rats (n = 3-4 per line) and monitored for over 30 days. Only SKOV3.ip1 produced reliable tumor engraftment; TOV21G, OVCAR3, and OVCAR8 failed to engraft at either site (data not shown). SKOV3.ip1 cells were resuspended to a density of 2 × 10^7^ cells per mL of medium. A total of 1 × 10^7^ SKOV3.ip1 cells per site were injected intraperitoneally (i.p.) into the lower left peritoneal quadrant and subcutaneously (s.c.) in the right flank using a 25-gauge needle. Non-tumor-bearing control rats received equivalent volume injections of vehicle (500 μL RPMI 1640 + 10% FBS) on the same sites.

Subcutaneous tumor growth was monitored twice weekly using a digital caliper, and tumor volumes were calculated using the ellipsoid volume formula (V = $\frac{\pi }{6}$ × L × W × H) (Tomayko and Reynolds, 1989). Intraperitoneal tumor establishment was confirmed at necropsy at the conclusion of the study. Survival time was defined as the interval from the day of SKOV3.ip1 implantation to euthanasia at the study endpoint or when humane endpoint criteria were met in accordance with IACUC guidelines (subcutaneous tumor diameter ≥ 3.5 cm, ≥ 20% body weight loss, or signs of emaciation).

Dual-site implantation enabled longitudinal assessment of tumor burden via subcutaneous measurements while maintaining clinically relevant intraperitoneal disease. This approach was selected to minimize reliance on repeated bioluminescent imaging with a luciferase-tagged line, which requires anesthesia and may confound systemic cytokine profiling and cognitive outcomes in preclinical models (Xu et al., 2023; Shen et al., 2013).

### 2.3 Ovarian tumor engraftment, monitoring, and pre-defined exclusion criteria

Tumor engraftment was defined by sustained, measurable subcutaneous tumor growth and/or macroscopic intraperitoneal disease confirmed at necropsy, characterized by vascularized and necrotic tissue, consistent with previously published gross pathological characterization of this model (Lomeli et al., 2025). Animals were monitored for evidence of spontaneous tumor regression, defined as a loss of measurable subcutaneous tumor burden in the absence of cisplatin treatment. Animals that failed to develop sustained and measurable subcutaneous tumors or intraperitoneal tumors, or that exhibited spontaneous tumor regression prior to cisplatin initiation, were excluded from the subcutaneous tumor volume and survival analyses (Fig. 1). Behavioral and cytokine analyses were performed in animals that received SKOV3.ip1 injections, regardless of persistent measurable tumor burden, as the primary objective of these endpoints was to assess the effects of ovarian tumor cell injection and cisplatin exposure on cognitive and inflammatory outcomes independent of persistent tumor burden.

### 2.4 Cisplatin administration

Cisplatin treatment was initiated two weeks following tumor inoculation. Animals received cisplatin (CDDP; 5 mg/kg, i.p; Teva Pharmaceuticals USA, Inc.) or an equal volume of 0.9% saline, i.p., once every two weeks for a total of 4 treatment cycles. To minimize renal toxicity and promote diuresis, all animals received mannitol (125 mg/kg, i.p.; APP Pharmaceuticals) 1 h before each CDDP injection. Because dehydration is a common adverse effect of CDDP treatment, animals were monitored for signs of dehydration using skin tenting assessments. Subcutaneous fluids (5-10 mL of 0.9% saline, s.c.) were administered as needed. Additionally, softened food pellets or DietGel® Recovery were provided in each cage as supportive care.

### 2.5 Behavioral testing

The behavioral assessments, including the open field test (OFT), context object discrimination (COD), and novel object recognition (NOR), were performed using established protocols with minor modifications, as previously described (Lomeli et al., 2017; Lomeli et al., 2024; Lomeli et al., 2025). After a 4-week rest period following the fourth CDDP treatment cycle, cognitive testing was conducted over approximately 3 weeks (4 to 7 weeks after the final CDDP cycle). Behavioral experiments were conducted across three independent animal cohorts, and data from all experimental groups were pooled for analysis, except for the OFT, which was conducted on only one cohort. Previously published data from Lomeli et al. (2025), collected from NT + VEH, NT + CDDP, OvT + VEH, OvT + CDDP, were incorporated into the present study to enable cross-task comparisons of cognitive outcomes (Fig. 2, Fig. 4). All behavioral testing was recorded using an overhead video camera (Swann Security System) and scored manually by an experimenter blinded to treatment conditions. Animals that explored for less than 5 seconds per object were excluded from the COD and NOR analysis.

**Fig. 2:**
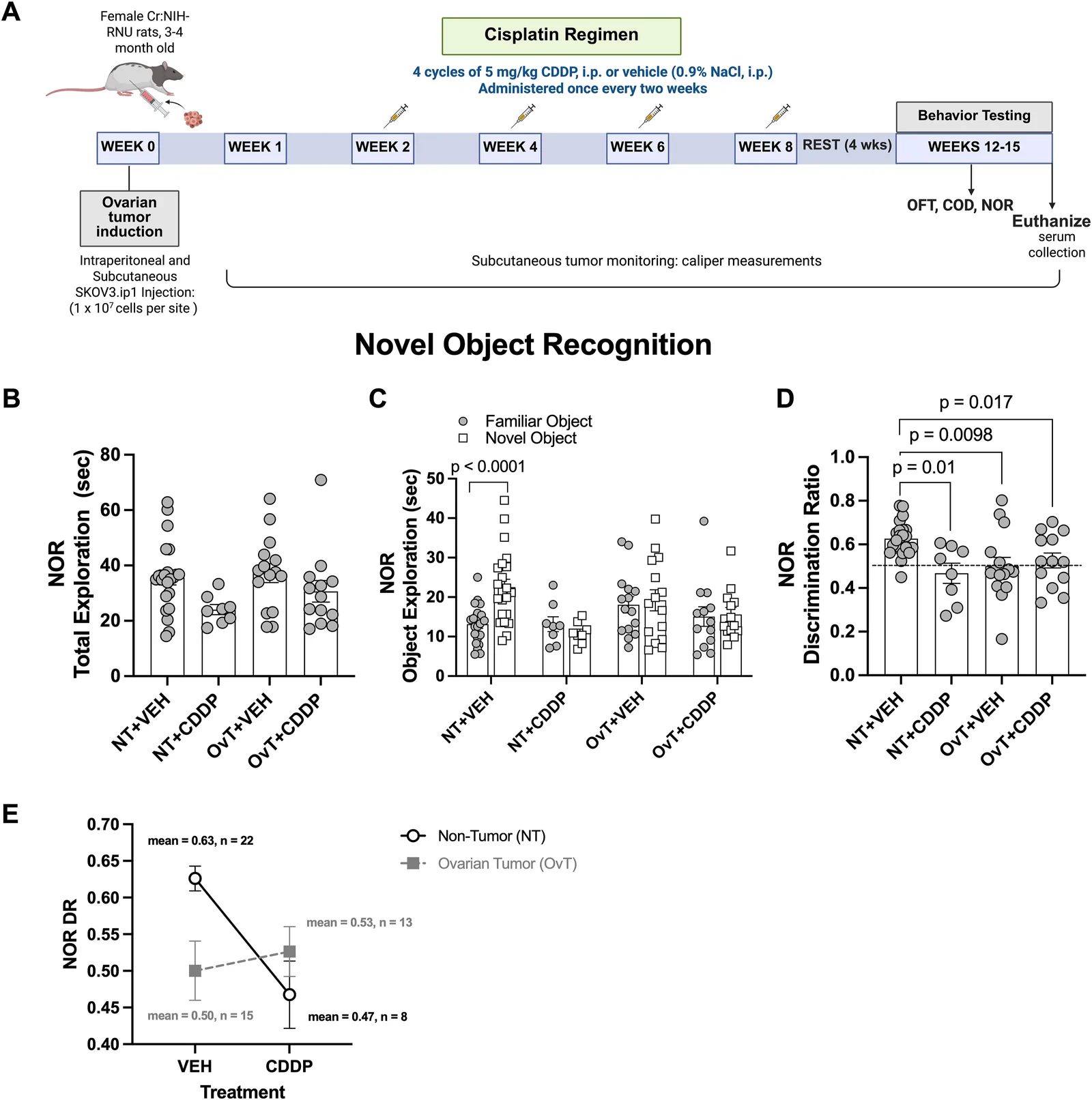
**Behavioral experimental design and assessment of cognitive function in ovarian tumor-bearing rats treated with cisplatin.** (**A**) Experimental timeline. Female RNU rats were implanted with 1 × 10^7^ SKOV3.ip1 cells (subcutaneous [s.c.] and intraperitoneal [i.p.]). Cisplatin (CDDP; 5 mg/kg, i.p.) or vehicle (0.9% NaCl, i.p.) was administered every 2 weeks for four cycles, beginning 2 weeks post-implantation. Following a 4-week rest period after the final CDDP cycle, behavioral testing (OFT, COD, NOR) was conducted over approximately 3 weeks (weeks 12-15 post-implantation). Serum was collected, and animals were euthanized at the end of testing (7 weeks after the final CDDP cycle) or earlier if humane endpoint criteria were met. **(B-E)** NOR: (**B**) total object exploration time, (**C**) time spent exploring familiar vs. novel objects, and (**D**) discrimination ratio (novel object exploration/total object exploration). (**E**) Novel object recognition discrimination ratio mean values are shown by treatment (VEH vs. CDDP) and tumor status (NT vs. OvT). Data are presented as mean ± SEM; each point represents one animal (**B-E**). Analysis of NOR discrimination ratios was performed using multiple regression with simple effects assessed by independent-group *t*-tests assuming heterogeneous variances. Abbreviations: CDDP, cisplatin; COD, context object discrimination; DR, discrimination ratio; NOR, novel object recognition; NT, non-tumor-bearing; OvT, ovarian tumor-bearing; VEH, vehicle.

#### 2.5.1 Open field test (OFT)

OFT was performed four weeks after completion of cisplatin to evaluate locomotor activity and anxiety-like behavior associated with ovarian cancer and chemotherapy exposure. Testing was conducted in a square Plexiglas arena (60 cm × 60 cm) with 60 cm-high walls, which were externally covered with opaque paper to minimize visual distractions. Rats were placed individually in the center of the arena and allowed to explore freely for 5 min. After each session, animals were returned to their home cages, and the arena was thoroughly cleaned with 70% ethanol and dried before subsequent testing. For the analysis, the arena was divided into a central zone (30 cm × 30 cm) and a surrounding peripheral zone. The total time spent in the center was measured; reduced exploration of the center compared with the peripheral zone was interpreted as increased anxiety-like behavior.

#### 2.5.2 Context object discrimination (COD)

COD testing was conducted using two distinct arenas (designated A and B) located in separate adjacent rooms, as previously described (Lomeli et al., 2017). For training, each arena contained two identical copies of the same object, but the objects were different between arenas. During training sessions, rats were exposed to each arena for 5 min per day, on two consecutive days, with a 20-min intertrial interval, and were then returned to their home cages. The order in which the arenas were experienced first was counterbalanced across animal and training days. On the test day, rats were placed in arena A, in which one of the original objects had been replaced by an object previously encountered only in arena B. Behavioral measures included total exploration time, time spent exploring each object, and the discrimination ratio, which is defined as the time spent exploring the out-of-context object divided by the total exploration time (in-context object + out-of-context object).

#### 2.5.3 Novel object recognition (NOR)

NOR testing was conducted in the same opaque Plexiglas arena used for OFT. During the training phase, rats were allowed to explore the arena containing two identical objects for 5 min per day over two consecutive days. The test session was performed 24 h after the final training session. For the test session, one familiar object was replaced with a novel object. Rats were allowed to explore for 5 min. Outcome measures included total object exploration time, time spent investigating each object, and the discrimination ratio (time spent investigating the novel object divided by total exploration time).

### 2.6 Serum cytokine profiling

Blood was collected via cardiac puncture into uncoated 1.5 mL tubes at 7 weeks post-CDDP completion (at euthanasia) and allowed to clot for 30 min at room temperature. The tubes were centrifuged at 2000 × g for 15 min. Serum was transferred into new 1.5 mL tubes and stored at −80°C until analysis. Serum from four randomly selected animals in each group (NT + VEH, NT + CDDP, OvT + VEH, OvT + CDDP) was sent to Quansys Biosciences for cytokine analysis.

The cytokines were assayed on Quansys Biosciences' (Logan, UT) Q-Plex™ Rat Cytokine Inflammation multiplexed ELISA array. The full cytokine panel and correlation results with NOR performance are presented in Supplementary Table 1. Samples were analyzed at the following dilution factors: 1:2, 1:20, and 1:200. Each dilution was measured in triplicate. All cytokines were detectable, except for IFN-γ, which is known to be reduced in RNU rats (Hougen, 1991). C-reactive protein (CRP) was quantified separately using a single-analyte enzyme-linked immunosorbent assay (ELISA) per the manufacturer's instructions (Cat # ERCRP; Thermo Fisher Scientific, Waltham, MA, USA). All available serum samples (5 μL per sample) were analyzed at a 1:20,000 dilution. The absorbance for the CRP assay was measured at 405 nm, and CRP was quantified by linear regression using validated CRP standards (R^2^ > 0.95, 0.312 – 60 ng/mL). All analyses were performed in duplicate with appropriate negative and positive controls.

### 2.7 Statistical analysis

Sample sizes for behavioral testing were informed by a prior power analysis using effect sizes derived from pooled data from our previous studies in non-tumor-bearing, cisplatin-treated rats, which differed from the current model in sex, age, and immune status (immunocompetent female Sprague Dawley retired breeders, 9 months (Lomeli et al., 2024); immunocompetent male Sprague Dawley, 3-4 months (Lomeli et al., 2017). Pooled analysis of these datasets indicated an approximate 16-17% reduction in NOR discrimination associated with CDDP treatment (Cohen's d ≈ 0.9), corresponding to approximately 20-24 animals per group to detect an effect of this magnitude with 80% power at α = 0.05 (two-tailed, independent samples *t*-test). Group sizes for non-tumor-bearing animals (NT + VEH: n = 24; NT + CDDP: n = 10) were allocated accordingly, with NT + CDDP treated as a confirmatory replicate of a previously characterized effect. Tumor-bearing groups (OvT + VEH, OvT + CDDP) were oversampled relative to the power analysis requirements for behavioral testing to offset anticipated attrition from incomplete tumor engraftment and humane endpoints.

Data are presented as mean ± SEM, and statistical significance was set at α = 0.05. For OFT, data were square-root-transformed to address right-skewness (skew = 1.6) and analyzed by multiple regression, assessing the effects of tumor status, CDDP treatment, and their interaction. To assess the effects of tumor cell implantation and CDDP treatment on mean COD and NOR discrimination ratio scores, data were analyzed using multiple regression with simple-effects analyses assessed by independent-groups *t*-tests, assuming heterogeneous variances. Normality of COD and NOR discrimination ratios was assessed using the Shapiro-Wilk test. Serum cytokine levels were analyzed by two-way ANOVA (tumor status × CDDP treatment). Where a significant interaction was observed, simple effects were assessed using independent-groups *t*-tests, assuming heterogeneous variances (Fig. 3, Supplementary Fig. 3).

**Fig. 3:**
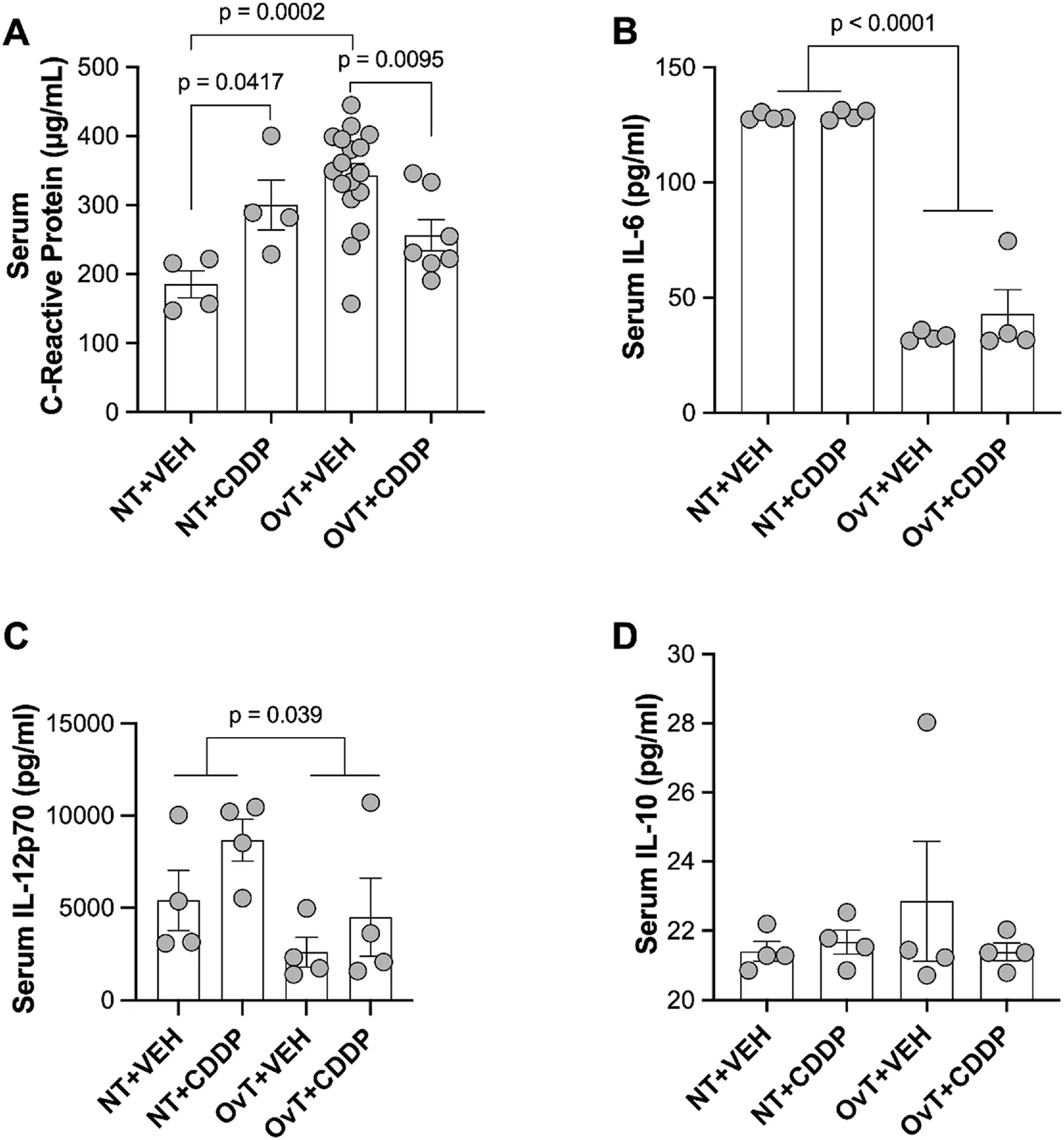
**Effects of tumor status and cisplatin treatment on circulating cytokine levels.** Serum was collected 7 weeks after completion of cisplatin. Serum levels of (**A**) C-reactive protein (CRP; μg/mL), (**B**) IL-6, (**C**) IL-12p70, and (**D**) IL-10 (pg/mL) are shown for non-tumor-bearing (NT) and ovarian tumor-bearing (OvT) animals treated with vehicle (VEH) or cisplatin (CDDP). Data are presented as mean ± SEM. Sample sizes were n = 4 per group for IL-6, IL-12p70, and IL-10; CRP sample sizes were NT + VEH: n = 4, NT + CDDP: n = 4, OvT + VEH: n = 17, OvT + CDDP: n = 7. Data were analyzed by two-way ANOVA (tumor status × CDDP treatment); simple effects were assessed by independent-groups *t*-tests when a significant interaction was observed.

**Fig. 4:**
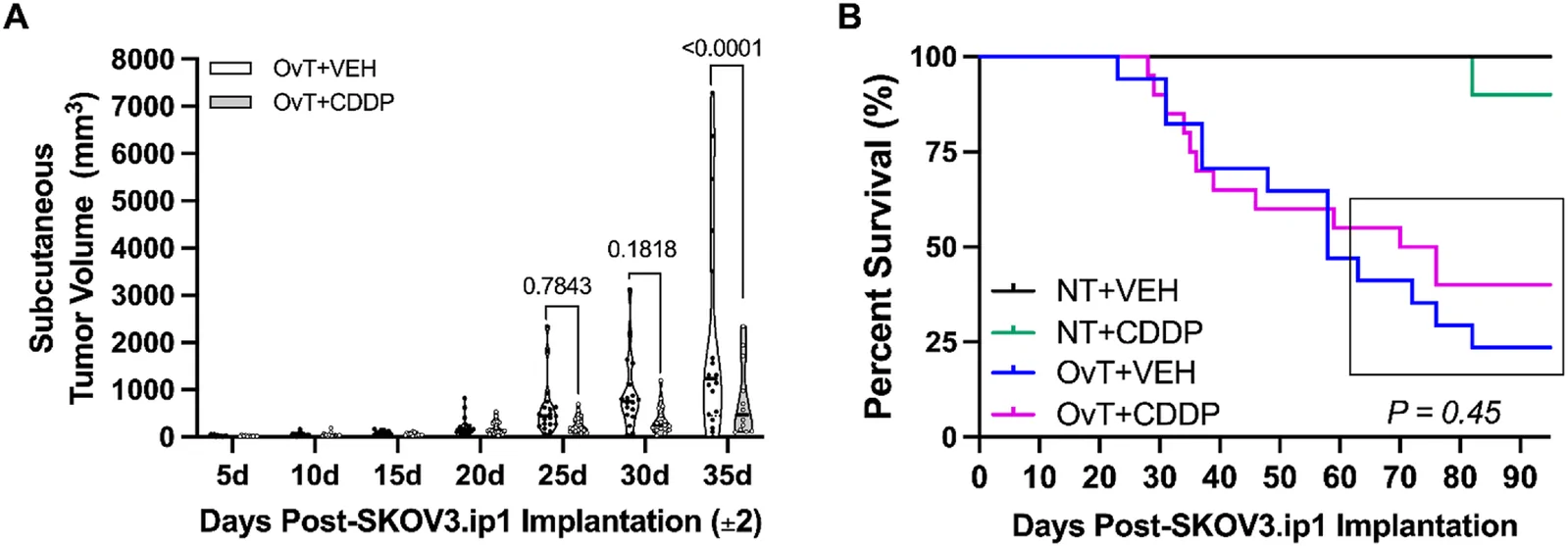
**Subcutaneous tumor growth and survivorship in the ovarian cancer rat model**. (**A**) Longitudinal measurement of subcutaneous tumor volume in ovarian tumor-bearing rats following SKOV3.ip1 implantation. Cisplatin (5 mg/kg, i.p.) was initiated two weeks post-implantation and administered every other week for four cycles. CDDP-treated animals (OvT + CDDP) showed reduced subcutaneous tumor burden compared with vehicle-treated controls (OvT + VEH). Data are presented as mean ± SEM and analyzed using a mixed-effects model followed by Sidak's multiple comparisons test. Representative measurements are shown at approximately 5-day intervals. (**B**) Kaplan-Meier survival curves for non-tumor-bearing and ovarian tumor-bearing animals treated with vehicle or CDDP.

Correlations between circulating cytokine levels and NOR discrimination ratio scores were estimated by Spearman's method to avoid the assumptions of normality and linearity. For exploratory biomarker analyses, continuous NOR discrimination ratio scores were dichotomized into impaired (≤0.5) and non-impaired (>0.5) groups. The potential of individual cytokines and serum C-reactive protein (CRP) to discriminate between these groups was assessed using receiver operating characteristic (ROC) curve analysis, with discrimination quantified by the area under the curve (AUC), sensitivity, and specificity. Survival was evaluated using the Kaplan-Meier curve, stratified by treatment group, and differences between groups were assessed using the log-rank test.

Behavioral and cytokine group comparisons were performed using GraphPad Prism 10; correlation, ROC, and multivariate modeling analyses were performed using SAS 9.4 (SAS Institute, Cary, NC). Figures were generated using GraphPad Prism 10 and BioRender.

## 3 Results

### 3.1 Tumor engraftment and animal inclusions

SKOV3.ip1 ovarian tumor engraftment was assessed at both subcutaneous (s.c.) and intraperitoneal (i.p.) sites across three independent experimental cohorts (Fig. 1). All non-tumor-bearing animals (NT + VEH, n = 24; NT + CDDP, n = 10) met the inclusion criteria and were retained for subsequent analyses. Among tumor-implanted animals, successful tumor establishment at one or both implantation sites was observed in 63% of OvT + VEH animals (n = 17) and 74% of OvT + CDDP animals (n = 20). Within the tumor-bearing cohorts, subcutaneous-only engraftment (IP-/SC+) was observed in 59% of OvT + VEH animals and 85% of OvT + CDDP animals. Dual-site engraftment (IP+/SC+) was observed in 41% and 10% of animals, respectively. Intraperitoneal-only engraftment (IP+/SC-) was uncommon, occurring in 0% of OvT + VEH and 5% of OvT + CDDP animals. A subset of animals was excluded from the subcutaneous tumor volume and survival analyses due to failure to establish tumors at either site or spontaneous regression of previously established subcutaneous tumors. Spontaneous regression accounted for most exclusions in the OvT + VEH group (80%, n = 8) and a portion of exclusions in the OvT + CDDP group (43%, n = 3). Animals excluded from the subcutaneous tumor volume and survival analyses were retained for the behavioral and cytokine analyses, per predefined criteria. This approach allowed assessment of long-term cognitive and inflammatory outcomes following tumor implantation and cisplatin or vehicle exposure, independent of persistent measurable tumor burden.

### 3.2 Behavioral outcomes

Anxiety-like behavior and cognitive function were evaluated four to seven weeks following completion of the final CDDP treatment cycle (Fig. 2A).

#### 3.2.1 Open field test (OFT)

Anxiety-like behavior was assessed using the open field test (OFT). No significant differences were observed among groups in time spent in the center zone (Supplementary Fig. 1); ovarian tumor implantation: *t*_*(*23)_ = 0.58, *p* = 0.57; CDDP treatment: *t*_(23)_ = 0.30, *p* = 0.77; interaction: *t*_(23)_ = −0.33, *p* = 0.75.

#### 3.2.2 Context object discrimination (COD)

Next, hippocampal-dependent contextual discrimination was assessed using the context object discrimination (COD) task. Total object exploration did not differ between groups (*p* = 0.75, Supplementary Fig. 2A). At the individual group level, NT + VEH (*t*_(22)_ = 3.23, *p* = 0.0039) and OvT + VEH (*t*_(13)_ = 2.93, *p* = 0.0118) animals showed a significant preference for the out-of-context object; no significant preference was observed in NT + CDDP (*t*_(7)_ = 1.25, *p* = 0.253) or OvT + CDDP (*t*_(11)_ = 0.04, *p* = 0.970) animals (Supplementary Fig. 2B). However, analysis of the COD discrimination ratio using multiple regression with simple effects assessed by independent-group *t*-tests, assuming heterogeneous variances, showed no significant tumor status × CDDP interaction or simple effects of tumor status or CDDP treatment (Supplementary Fig. 2C).

#### 3.2.3 Novel object recognition (NOR)

To further assess recognition memory, which relies primarily on the perirhinal cortex, with additional contributions from the hippocampus (Broadbent et al., 2010; Kinnavane et al., 2016), animals were tested using the novel object recognition (NOR) task. During the test phase, total object exploration did not differ between groups (Fig. 2B). NT + VEH rats demonstrated intact recognition memory, spending significantly more time exploring the novel object than the familiar object (Fig. 2C; *t*_(21)_ = 5.77, *p* < 0.0001), whereas no significant preference was observed in NT + CDDP (*t*_(7)_ = 0.93, *p* = 0.384), OvT + VEH (*t*_(14)_ = 0.34, *p* = 0.740), or OvT + CDDP (*t*_(12)_ = 0.28, *p* = 0.783; Fig. 2C). Analysis of the NOR discrimination ratios was performed using multiple regression, with simple effects assessed by independent-groups *t*-tests (Fig. 2D and E). A significant interaction between tumor status and CDDP treatment (*t*_(54)_ = 2.75, *p* = 0.008) was detected, indicating that the effect of CDDP on NOR performance differed depending on tumor status. CDDP treatment was associated with a marked decline in discrimination ratios in NT + CDDP animals compared with NT + VEH (*t*_(8.96)_ = 3.25, *p* = 0.01). OvT + VEH animals showed significantly lower discrimination ratios than NT + VEH animals (*t*_(18.88)_ = 2.87, *p* = 0.0098), indicating that tumor implantation alone was associated with reduced discrimination performance in the absence of CDDP. OvT + CDDP animals also showed significantly lower discrimination ratios than NT + VEH (*t*_(17.96)_ = 2.63, *p* = 0.017). No significant difference in discrimination ratios was observed between OvT + VEH and OvT + CDDP (*t*_(25.77)_ = 0.49, *p* = 0.63). Together, these results indicate that NT + VEH was the only group with intact discrimination performance; NT + CDDP, OvT + VEH, and OvT + CDDP were each significantly impaired relative to NT + VEH but did not differ significantly from one another, consistent with a floor effect once discrimination was impaired by either tumor presence or CDDP treatment.

### 3.3 Systemic cytokine profiles post-chemotherapy

Serum cytokine levels were assessed seven weeks following completion of cisplatin treatment to evaluate chronic systemic inflammatory changes associated with ovarian tumor burden and chemotherapy exposure. Several cytokines, including C-reactive protein (CRP), IL-1α, IL-4, IL-6, TNF-α, and IL-12p70, differed significantly across experimental groups (Fig. 3, Supplementary Fig. 3), indicating persistent alterations in immune signaling**.**

Serum CRP levels showed a significant tumor status × CDDP interaction (*F*_(1,28)_ = 13.04, *p* = 0.0012), indicating that CDDP had opposite effects on CRP depending on tumor status (Fig. 3A). Within non-tumor-bearing animals, CDDP exposure increased CRP levels relative to NT + VEH (*t*_(4.61)_ = 2.80, *p* = 0.0417). In contrast, within tumor-bearing animals, CDDP reduced CRP levels relative to OvT + VEH (*t*_(13.35)_ = 3.03, *p* = 0.0095). Tumor implantation itself was associated with elevated CRP within vehicle-treated animals (NT + VEH vs. OvT + VEH: *t*_(8.66)_ = 6.05, *p* = 0.0002), but tumor status had no significant effect within CDDP-treated animals (NT + CDDP vs. OvT + CDDP: *t*_(5.43)_ = 1.03, *p* = 0.347).

Several cytokines showed a significant main effect of tumor status, independent of CDDP treatment and with no significant tumor × CDDP interaction: IL-6 (*F*_(1,12)_ = 289.3, *p* < 0.0001), TNF-α (*F*_(1,12)_ = 65.03, *p* < 0.0001), IL-4 (*F*_(1,12)_ = 38.8, *p* < 0.0001), IL-1α (*F*_(1,12)_ = 15.1, *p* = 0.0022), and IL-12p70 (*F*_(1,12)_ = 5.4, *p* = 0.039). IL-6, TNF-α, and IL-12p70 were reduced in tumor-bearing animals relative to non-tumor-bearing controls (Fig. 3B and C; Supplementary Fig. 3A), while IL-1α and IL-4 were increased in tumor-bearing animals (Supplementary Fig. 3B and C), indicating a tumor-associated shift in cytokine signaling.

No significant main effects of tumor status or CDDP treatment, or a significant interaction, were observed for IL-1β, IL-2, or IL-10 (Fig. 3D, Supplementary Fig. 3D-E). IL-10 levels showed a modest, non-significant increase following CDDP treatment in both non-tumor-bearing and tumor-bearing groups and were further examined in relation to behavioral outcomes. IFN-γ levels were uniformly low across all groups, consistent with its expected absence in this athymic model and supporting its value as an internal negative control (Supplementary Fig. 3F).

Together, these findings demonstrate persistent alterations in systemic cytokine profiles following tumor implantation and CDDP treatment. Given the observed impairments in recognition memory, these changes in circulating immune signaling may be associated with cognitive dysfunction in this model.

### 3.4 Correlation between cytokines and cognition

To investigate whether the alterations in circulating cytokines were associated with cognitive outcomes, we assessed the correlations between serum cytokine levels and the NOR discrimination ratio using Spearman's rank correlation (Supplementary Table 1). Correlation coefficients (r_s_) between cytokine levels and the NOR discrimination ratio were predominantly negative, though individual correlations did not reach statistical significance. Based on the magnitude of observed correlations, four cytokines (IL-6, IL-10, IL-12p70, and CRP) with an absolute value of r_s_ > 0.2 were selected for further exploratory analysis. Scatter plots of NOR discrimination ratio scores against cytokine levels are presented in Supplementary Fig. 4. To evaluate whether these cytokines could distinguish between impaired and non-impaired animals, NOR discrimination ratios were dichotomized into low (≤0.5) and high (>0.5) performance groups, and their ability to differentiate between these groups was assessed using the area under the curve (AUC; Table 1). IL-10 demonstrated the highest AUC (0.765), followed by CRP (0.699), whereas IL-6 and IL-12p70 showed lower discriminative ability. CRP and IL-10 were not correlated (r_s_ = −0.16, *p* = 0.61, n = 13), suggesting that they make independent contributions to classification performance.

**Table 1:** **Area Under Curve (AUC) analysis of serum cytokines for distinguishing cognitive status**. Novel object recognition (NOR) discrimination ratios were dichotomized into low (≤0.5) and high (>0.5) performance groups to define impaired and non-impaired groups. AUC was used to assess the ability of each cytokine to distinguish between the two groups. Sample size (n), AUC, and the 95% confidence interval are shown for each cytokine.

Area Under Curve for Cytokines Predicting NOR Discrimination Status				
Cytokine	n	AUC	95% CI	95% CI
			Lower	Upper
IL-6	14	0.643	0.319	0.967
IL-10	14	0.765	0.496	1.000
IL-12p70	14	0.571	0.236	0.906
CRP	25	0.699	0.482	0.916

To further explore the combined effects of selected cytokines associated with NOR discrimination performance, multivariate models were developed using IL-6, IL-10, IL-12p70, and CRP as candidate covariates. Given the correlation between IL-6 and IL-12p70 (r_s_ = 0.50, *p* < 0.05, n = 16; Supplementary Table 2), IL-12p70 was excluded from multivariate models in favor of IL-6, which demonstrated a stronger univariate AUC (Table 1). The resulting candidate models therefore included IL-6, IL-10, and CRP in two combinations: IL-6 with IL-10 and IL-10 with CRP. A third model combining IL-6 and CRP was also tested but was excluded, as the small sample (n = 11) allowed these markers to perfectly separate the impaired and non-impaired groups, producing an unreliable model fit. Key metrics for the resulting models are shown in Table 2. Among the models evaluated, the combination of IL-10 and CRP demonstrated the highest classification performance (AUC = 0.867) and the lowest Akaike Information Criterion (AIC), demonstrating improved model fit relative to the IL-6/IL-10 model. However, the small sample sizes available for these models (n = 14 for IL-6/IL-10; n = 11 for IL-10/CRP) limit interpretation of this exploratory analysis. Together, these findings suggest that IL-10 and CRP may jointly contribute to cognitive performance in this model and represent candidate circulating markers for further investigation.

**Table 2:** **Comparison of multivariate models incorporating cytokines associated with novel object recognition task performance.** Comparison of multivariate models evaluating combinations of serum cytokines as covariates for distinguishing impaired (NOR discrimination ratio ≤ 0.5) vs. non-impaired (NOR discrimination ratio > 0.5) animals. Models were assessed using the area under the curve (AUC), the lower bound of the 95% confidence interval, the Akaike information criterion (AIC), and overall model significance (test of whether all slopes are equal to zero). Sample size (n) is shown for each model.

Comparison of Metrics for Multivariate Models						
Model	Covariates	AUC	Lower Bound 95% CI	AIC	Model p-value	n
1	IL-6 IL-10	0.765	0.496	21.684	0.1553	14
2	IL-10 CRP	0.867	0.635	16.095	0.0795	11

### 3.5 Tumor growth and survival

CDDP treatment was initiated two weeks following SKOV3.ip1 tumor cell implantation, at which time subcutaneous tumors were detectable by digital caliper measurement (Fig. 4A). Subcutaneous tumor growth was monitored longitudinally, while intraperitoneal tumor establishment was confirmed at necropsy. Consistent with our prior description of the SKOV3.ip1 ovarian cancer model, intraperitoneal tumors disseminated throughout the peritoneal cavity and displayed gross morphological features characteristic of human ovarian carcinoma (Lomeli et al., 2025). CDDP treatment significantly reduced subcutaneous tumor burden in ovarian tumor-bearing rats compared with vehicle-treated controls (OvT + CDDP vs. OvT + VEH; *F*_(6,167)_ = 3.599, *p* = 0.0022; Fig. 4A). Overall survival over the study duration did not differ significantly between treatment groups (log-rank test, *p* = 0.45; Fig. 4B). Median survival was 58 days for OvT + VEH (n = 17) and 73 days for OvT + CDDP (n = 20). One non-tumor-bearing rat in the NT + CDDP group completed all four cycles of CDDP treatment but reached the predefined humane endpoint (20% body weight loss) and was euthanized in accordance with IACUC guidelines (Fig. 4B).

Long-term survivors were defined as ovarian tumor-bearing rats that completed all four treatment cycles and survived through the completion of novel object recognition (NOR) testing. The presence of long-term survivors in both OvT + VEH and OvT + CDDP groups enabled evaluation of persistent cognitive and inflammatory outcomes following tumor implantation and chemotherapy exposure.

## 4 Discussion

This study, to our knowledge, presents the first rat ovarian cancer model of CRCI that integrates a clinically relevant cisplatin regimen with behavioral outcomes and systemic cytokine analysis. To date, cancer-specific CRCI models in rats remain limited, with most studies using mice or non-tumor-bearing models. In ovarian cancer specifically, rat models are scarce; existing approaches include chemically induced models (e.g., DMBA) and the spontaneous ovarian carcinoma model in Lewis rats (Ribeiro de Souza et al., 2025; Sharrow et al., 2010). Building on our prior work in non-tumor-bearing rat models, we developed a human SKOV3.ip1 xenograft model in female athymic nude RNU rats to enable investigation of tumor-treatment interactions and potential therapeutic strategies for CRCI.

The selection of SKOV3.ip1 cell line warrants comment. Although SKOV3.ip1 is widely used and known to engraft reliably and metastasize consistently in immunodeficient murine models, it diverges from the typical genomic and transcriptomic profile of high-grade serous ovarian cancer (HGSOC) in large-scale comparisons with patient tumor data (Domcke et al., 2013), reflecting a broader trade-off documented in the field. Cell lines with closer genomic fidelity to HGSOC, such as KURAMOCHI and OVSAHO, engraft poorly as xenografts, particularly at subcutaneous sites, whereas SKOV3 reliably engrafts across multiple xenograft models (Elias et al., 2015). Given that establishing a feasible, reproducible ovarian cancer rat model was the primary goal of this study, SKOV3.ip1 was used for this purpose, with cell lines of greater HGSOC fidelity representing a valuable extension for future work.

Tumor burden has been associated with systemic biological changes, including inflammatory signaling, that may contribute to cognitive dysfunction and altered susceptibility to chemotherapy-induced neurotoxicity. In the present study, both OvT + VEH and OvT + CDDP animals showed significantly lower NOR discrimination than NT + VEH animals (*p* = 0.0098 and *p* = 0.017, respectively), while OvT + VEH and OvT + CDDP did not differ from each other, indicating that tumor implantation alone was associated with cognitive deficits in the absence of CDDP treatment. These findings further indicate that the interaction between peripheral tumor burden and chemotherapy is complex. Cisplatin-induced impairment in novel object recognition was most evident in non-tumor-bearing animals, whereas tumor-bearing animals exhibited lower baseline discrimination with minimal behavioral changes following cisplatin treatment. One potential explanation is that cisplatin may exert differential effects in tumor-bearing animals by targeting tumor cells, as evidenced by reduced subcutaneous tumor burden in the OvT + CDDP group, thereby modifying downstream neurotoxic effects compared with non-tumor-bearing animals. These findings raise the possibility that therapeutic intervention prior to the accumulation of substantial tumor burden may limit tumor-associated neuroinflammatory signaling and thereby reduce a key driver of cognitive dysfunction, supported by the observed reduction in serum CRP in the OvT + CDDP group. Whether this reflects durable improvement or effects specific to this single post-treatment timepoint remains an open question, particularly given clinical reports of persistent CRCI after chemotherapy completion (Janelsins et al., 2014).

CDDP treatment did not significantly affect COD discrimination, whereas NOR discrimination showed a significant tumor status × CDDP interaction, reflecting a divergence between the two tasks in their sensitivity to tumor and CDDP status. Within NOR, this interaction was driven by a CDDP-associated decline confined to non-tumor-bearing animals; no significant CDDP effect was observed within the OvT groups, which instead showed a small, non-significant increase in discrimination ratio. The task-level divergence between COD and NOR may reflect differences in the underlying neural circuits or task demands, as COD relies primarily on hippocampal, context-dependent discrimination, whereas NOR depends primarily on perirhinal cortex-mediated, novelty-based recognition memory (Broadbent et al., 2010; Kinnavane et al., 2016). Separately, a further contributing factor within NOR may be limited assay sensitivity: OvT animals already showed discrimination ratios near chance level at baseline (OvT + VEH, NOR DR = 0.50), and due to the sensitivity of the assay, we may not have been able to detect a further CDDP-associated decrease, consistent with the absence of a further decline in the OvT + CDDP group. This pattern is consistent with tumor cell implantation itself partially impairing NOR performance in the OvT + VEH group, potentially masking an additive effect of CDDP.

To our knowledge, no ovarian cancer rat model of CRCI had previously been established; preclinical CRCI models have relied primarily on non-tumor-bearing paradigms or mouse tumor-bearing models. A rat model was selected to build on our prior characterization of cisplatin-induced cognitive impairment in non-tumor-bearing rats (Lomeli et al., 2017, Lomeli et al., 2024), allowing us to extend established behavioral paradigms and methodology to a tumor-bearing context. We used this model to evaluate the neuroprotective efficacy of NAC in a tumor-bearing model (Lomeli et al., 2025). The lack of well-characterized syngeneic rat ovarian cancer cell lines led us to select a xenograft-based approach using the human SKOV3.ip1 line, which required an immunodeficient host; athymic nude RNU rats were selected, remaining within the same species as our prior work. Although RNU rats lack functional T cells, they retain components of the innate immune system, including macrophages, natural killer cells, and B cells (Rolstad, 2001). The persistence of cognitive impairment and altered cytokine signaling in this T-cell-deficient model indicates that innate immune mechanisms alone are sufficient to produce measurable deficits, though the relative contribution of adaptive immunity, absent here, to the overall magnitude of these effects remains an open question for future studies in immunocompetent models.

Extending these behavioral observations, tumor implantation and cisplatin exposure were associated with alterations in systemic cytokine profiles, reflecting a complex, tumor-dependent immune response. Non-tumor-bearing animals unexpectedly displayed higher serum IL-6 and TNF-α levels than tumor-bearing animals, in contrast to the elevated CRP levels observed with tumor burden. This finding may reflect that CRP production is driven not only by IL-6 but also by IL-1 subfamily cytokines and TNF-α collectively (Bruserud et al., 2020), such that elevated CRP does not require concurrent elevation of any single upstream cytokine and may instead reflect upstream drivers not measured in our present panel. Tumor-associated systemic immunosuppression, including mechanisms such as myeloid-derived suppressor cell expansion, has also been shown to dampen circulating pro-inflammatory cytokine production despite persistent tumor burden and other markers of systemic inflammation (Umansky et al., 2016), and this may be further influenced by the athymic status of the RNU model. Since cytokines were measured using a rat-specific assay, tumor-derived human cytokines, including IL-6, which is constitutively secreted by the SKOV3.ip1 parental line (Watson et al., 1990), would not have been detected, potentially underestimating the total tumor-associated cytokine burden.

To further explore potential relationships between systemic inflammation and cognitive outcomes, cytokines were evaluated in relation to NOR performance. Based on correlation analyses, a subset of cytokines (CRP, IL-6, IL-10, and IL-12p70) was selected for exploratory modeling. Among these, CRP and IL-10 demonstrated the strongest ability to distinguish between impaired and non-impaired animals, although the overall discriminative performance was modest. Notably, CRP has been reported to be elevated in patients with ovarian cancer and is widely used as a marker of systemic inflammation, supporting its relevance in this disease context (Peres et al., 2019). CRP has also been associated with cognitive outcomes in breast cancer survivors, as well as in aging populations and in neurological disorders such as Alzheimer's disease, and more recently in depression (Tachibana et al., 2024; Noble et al., 2010; Chang et al., 2012; O'Bryant et al., 2010; Carroll et al., 2023). These findings should be interpreted with caution, given the limited sample size and the exploratory nature of the analyses. CRP and IL-10 in particular warrant further investigation as candidate markers, in future, larger studies of this model.

There are several limitations inherent to this model. Brain correlates, such as histology or region-specific cytokine expression, were not evaluated in this study and remain an area for future investigation. Animals completed all four cycles of cisplatin, with behavioral testing conducted approximately 4 to 7 weeks after treatment completion and serum collection, and euthanasia occurring at the end of this window (7 weeks post-treatment, or earlier if humane endpoint criteria were met), representing a single post-treatment window rather than the full temporal trajectory of cognitive and inflammatory changes; cognitive effects may differ at earlier stages of tumor progression or during active chemotherapy exposure.

The dual-site (intraperitoneal and subcutaneous) implantation approach, necessary for longitudinal tumor monitoring without repeated anesthesia, produced heterogeneous engraftment outcomes: dual-site engraftment occurred in only 41% of OvT + VEH and 10% of OvT + CDDP animals, with most tumor-bearing animals developing subcutaneous-only disease and intraperitoneal-only engraftment remaining rare. Given the sample size required for this study, further stratification of outcomes by engraftment subtype was not statistically feasible. As this study represents, to our knowledge, the first methodological characterization of this xenograft approach in rats, we report this heterogeneity explicitly, as a documented outcome of the model. Intraperitoneal tumor status was confirmed qualitatively at necropsy but was not quantitatively measured. Subcutaneous tumor volume therefore served as the only quantified longitudinal measure of tumor burden in this study. Retaining animals without confirmed persistent tumor burden in the behavioral and cytokine analyses, per our predefined inclusion criteria, allowed assessment of the effects of tumor cell injection broadly, consistent with findings that factors secreted by cancer cells may induce lasting cognitive and molecular effects independent of persistent tumor burden. Soluble factors secreted by the 4T1.2 epithelial breast cancer cell line, in the absence of live tumor cells, have been shown to be sufficient to induce cognitive impairment in mice (Walker et al., 2018), and spontaneous tumor regression has been shown not to reverse tumor-associated proteomic changes in the hippocampus, with regressed and actively tumor-bearing animals displaying similar altered hippocampal proteomes (Mampay et al., 2025). Our study is further constrained by the small sample size used for cytokine profiling (n = 4 per group), and the sample sizes (n = 11 -25) available for correlation and ROC analyses. Additionally, the effect size estimates used to inform sample size for behavioral testing were derived from immunocompetent Sprague Dawley rats differing in sex and age from the RNU model used here (Lomeli et al., 2017; Lomeli et al., 2024); given the immunocompetent status of these models, these estimates may not fully generalize to the present model, and achieved power for the pairwise NT + CDDP vs. NT + VEH comparison (n = 10 vs. n = 24) was approximately 56-64% for effect sizes of this magnitude. Future studies incorporating immunocompetent ovarian cancer models, cell lines with greater HGSOC fidelity, a broader cytokine panel with assessment of myeloid cell populations, larger cohorts, and longitudinal sampling will be needed to further validate and extend these findings.

In summary, this study establishes a clinically relevant rat tumor-bearing xenograft model of CRCI in ovarian cancer that enables the investigation of how chemotherapy and tumor biology jointly influence cognitive outcomes. These findings demonstrate that the cognitive effects of cisplatin differ depending on tumor status, underscoring the importance of incorporating tumor-bearing models into preclinical studies of CRCI. Moreover, integrating behavioral paradigms with systemic cytokine profiling provides a framework for identifying candidate inflammatory markers associated with cognitive function. Together, this model offers a translational platform for future mechanistic studies and the development of therapeutic strategies to mitigate CRCI in ovarian cancer.

## CRediT authorship contribution statement

**Naomi Lomeli:** Conceptualization, Data curation, Formal analysis, Investigation, Methodology, Project administration, Validation, Visualization, Writing – original draft, Writing – review & editing. **Diana C. Pearre:** Conceptualization, Data curation, Formal analysis, Investigation, Writing – review & editing. **Javier J. Lepe:** Data curation, Formal analysis, Investigation, Writing – review & editing. **Donovan A. Argueta:** Data curation, Formal analysis, Investigation, Writing – review & editing. **Mya A. Arellano:** Data curation, Formal analysis, Investigation, Writing – review & editing. **Kalpna Gupta:** Funding acquisition, Resources, Supervision, Writing – review & editing. **Thomas H. Taylor:** Conceptualization, Formal analysis, Validation, Writing – original draft, Writing – review & editing. **Daniela A. Bota:** Conceptualization, Data curation, Formal analysis, Funding acquisition, Project administration, Supervision, Visualization, Writing – original draft, Writing – review & editing.

## Ethics declaration

This study was conducted in accordance with the ARRIVE (Animal Research: Reporting of In Vivo Experiments) guidelines as well as the following guidelines for animal welfare and/or reporting: Guide for the Care and Use of Laboratory Animals: Eighth Edition. This study was approved by the IACUC at the University of California, Irvine. (IACUC Approval No. AUP-22-097)

## Funding

This work was supported by the 10.13039/100000065NINDS/NIH [NS072234], 10.13039/100006108NCATS/NIH [UL1 TR001414], aiand the 10.13039/100007345UCI Cancer Center Award [P30CA062203] from the 10.13039/100000054NCI/NIH to 10.13039/100016129DAB, and 10.13039/100000054NCI/NIH R01 [CA263806] to 10.13039/100016129DAB and KG. The 10.13039/100006108NCATS/10.13039/100000002NIH TL-1 training grant [5 TL1 TR 1415-8] supported NL. 10.13039/501100000957DCP was supported by 10.13039/100000066NIEHS/10.13039/100000002NIH [T32CA060396]. The 10.13039/100009350Giannini Foundation Fellowship, 10.13039/100005595UC President's Postdoctoral Fellowship, and 10.13039/100000002NIH MOSAIC K99 [AT012494] grant supported 10.13039/501100018941DAA. *The content is solely the authors' responsibility and does not necessarily represent the official views of the* 10.13039/100000002*National Institutes of Health**.*

## Declaration of competing interest

The authors declare that they have no known competing financial interests or personal relationships that could have appeared to influence the work reported in this paper.
