# Supplementary material for: A novel rat model of ovarian cancer and cisplatin chemotherapy-related cognitive impairment
Source: Brain Behav Immun Health. 2026 Sep 22;57:101373. doi: 10.1016/j.bbih.2026.101373 (PMC13635444; doi:10.1016/j.bbih.2026.101373)
Supplement: Supplementary file 1 [file mmc1.docx]

|  | Non-Tumor (NT) | | Ovarian Tumor (OvT) | |  |
| --- | --- | --- | --- | --- | --- |
|  | **VEH** | **CDDP** | **VEH** | **CDDP** | **Total** |
| N | 8 | 9 | 6 | 4 | 27 |
| Mean | 22.4 | 27.7 | 39.6 | 27.6 | 28.7 |
| Median | 20.2 | 23.2 | 37.2 | 28.3 | 22.5 |
| Min | 2.8 | 0.0 | 0.0 | 13.8 | 0.0 |
| Max | 45.9 | 91.1 | 92.9 | 40.1 | 92.9 |

**B**

**A**

**Supplementary Figure 1. Assessment of anxiety-like behavior in ovarian tumor-bearing rats treated with cisplatin.** (**A**) The open field test (OFT) was performed 4 weeks after the final CDDP cycle. Time (seconds) spent in the center zone over a 5-minute duration. Data are presented as mean ± SEM; each point represents one animal. (**B**) Table summarizing the time-in-center data from OFT by tumor-implant and treatment status. These data were highly right-skewed (skew = 1.6), which was corrected by a square-root transformation. Assessment of the effects of ovarian tumor implantation and CDDP administration was conducted by multiple regression on the transformed data. No statistically significant effects were observed (ovarian tumor implantation: *t_(_*_23)_ = 0.58, *p* = 0.57; CDDP treatment: *t*_(23)_ = 0.30, *p* = 0.77; interaction: *t*_(23)_ = -0.33, *p* = 0.75).

**Supplementary Figure 2. Assessment of hippocampal-dependent contextual discrimination in ovarian tumor-bearing rats treated with cisplatin.** (**A**) Total object exploration time. (**B**) Time spent exploring the in-context vs. out-of-context objects. (**C**) Discrimination ratio (out-of-context object exploration/total object exploration). Data are presented as mean ± SEM; each point represents one animal. To assess the effects of tumor cell implantation and CDDP treatment on COD discrimination, data were analyzed by multiple regression with simple effects assessed by independent-groups *t*-tests, assuming heterogeneous variances, as for NOR. No significant tumor status × CDDP interaction was observed (*p* = 0.78). No significant simple effect of CDDP treatment was observed within non-tumor-bearing (*t*_(13.02)_ = 0.90, *p* = 0.386) or tumor-bearing animals (*t*_(23.45)_ = 1.96, *p* = 0.062), nor was a significant simple effect of tumor status observed within vehicle-treated (*t*_(34.89)_ = 0.31, *p* = 0.756) or CDDP-treated animals (*t*_(11.23)_ = 0.59, *p* = 0.570).

**Supplementary Figure 3. Additional circulating cytokine profiles following tumor implantation and cisplatin treatment.** Serum cytokine levels of (**A**) TNF-α, (**B**) IL-1α, (**C**) IL-4, (**D**) IL-1β, (**E**) IL-2, and (**F**) IFN-γ (pg/mL) are shown for non-tumor-bearing (NT) and ovarian tumor-bearing (OvT) rats treated with vehicle (VEH) or cisplatin (CDDP), seven weeks post-cisplatin treatment completion. Data are presented as mean ± SEM. Data were analyzed by two-way ANOVA (tumor status × CDDP treatment); simple effects were assessed by independent-groups *t*-tests when a significant interaction was observed. IFN-γ was below the level of detection in RNU rats and was therefore excluded from correlation analyses.

| Cytokine | n | r_s_ | p |
| --- | --- | --- | --- |
| CRP | 25 | -0.22 | 0.28 |
| IL-1α | 14 | 0.06 | 0.83 |
| IL-1β | 14 | -0.11 | 0.71 |
| IL-2 | 14 | -0.13 | 0.67 |
| IL-4 | 14 | -0.10 | 0.73 |
| IL-6 | 14 | -0.23 | 0.44 |
| IL-10 | 14 | -0.32 | 0.26 |
| IL-12p70 | 14 | -0.32 | 0.26 |
| TNF-α | 14 | -0.16 | 0.57 |
| IFN-γ | - | - | - |

**Supplementary Table 1.** **Association of novel object recognition task discrimination ratios with serum cytokine levels.** Spearman correlation coefficients (r_s_) between serum cytokine levels and novel object recognition (NOR) discrimination ratios. Sample size (n) and corresponding *p*-values are shown for each cytokine. We selected the four cytokines with an absolute value of r_s_ > 0.2, C-reactive protein, IL-6, IL-10, and IL-12p70, for further analyses.

**Supplementary Figure 4. Correlation between circulating cytokines and novel object recognition task performance.** Scatter plots showing the relationship between serum cytokine levels of (**A**) C-reactive protein (CRP; µg/mL), (**B**) IL-6, (**C**) IL-10, and (**D**) IL-12p70 (pg/mL) and novel object recognition (NOR) discrimination ratio for individual animals. Each point represents one animal. The horizontal reference line corresponds to the discrimination ratio threshold (0.5) used to define impaired and non-impaired NOR performance. Associations were assessed using Spearman’s rank correlation.

Pairwise correlations between circulating cytokines

|  | IL-10 | IL-12 | CRP |
| --- | --- | --- | --- |
| IL-6 | **0.35 (p = 0.19, n =16)** | **0.50 (p < 0.05, n = 16)** | **-0.24 (p = 0.42, n = 13)** |
| IL-10 | **-----** | **0.31 (p = 0.25, n = 16)** | **-0.16 (p = 0.61, n = 13)** |
| IL-12 |  | **-----** | **0.18 (p = 0.57, n = 13)** |
| CRP |  |  | **-----** |

**Supplementary Table 2. Pairwise correlations between circulating cytokines.** Spearman correlation coefficients (r_s_), *p*-values, and sample sizes (n) are shown for each cytokine pair.
